# Gene Prioritization by Integrated Analysis of Protein Structural and Network Topological Properties for the Protein-Protein Interaction Network of Neurological Disorders

**DOI:** 10.1155/2016/9589404

**Published:** 2016-03-13

**Authors:** Yashna Paul, Yasha Hasija

**Affiliations:** Department of Biotechnology, Delhi Technological University, Shahbad Daulatpur, Main Bawana Road, New Delhi, Delhi 110042, India

## Abstract

Neurological disorders are known to show similar phenotypic manifestations like anxiety, depression, and cognitive impairment. There is a need to identify shared genetic markers and molecular pathways in these diseases, which lead to such comorbid conditions. Our study aims to prioritize novel genetic markers that might increase the susceptibility of patients affected with one neurological disorder to other diseases with similar manifestations. Identification of pathways involving common candidate markers will help in the development of improved diagnosis and treatments strategies for patients affected with neurological disorders. This systems biology study for the first time integratively uses 3D-structural protein interface descriptors and network topological properties that characterize proteins in a neurological protein interaction network, to aid the identification of genes that are previously not known to be shared between these diseases. Results of protein prioritization by machine learning have identified known as well as new genetic markers which might have direct or indirect involvement in several neurological disorders. Important gene hubs have also been identified that provide an evidence for shared molecular pathways in the neurological disease network.

## 1. Introduction

Seizures and comorbid conditions like anxiety, depression, and cognitive impairment are some of the shared symptoms in patients with neurological disorders. This observation implies that these neurological disorders have certain shared genetic markers and molecular pathways that lead to their common clinical manifestations. There might be genetic markers associated with one disease, the mutations in which result into over- or underexpression of associated genes and interconnected molecular pathways. Such aberrations can cause similar observable symptoms in patients with different neurological disorders. For example, there are reports that suggest that epilepsy occurs in approximately 8 to 20% of children with autism spectrum disorders with an increasing prevalence of seizures occurring in the late adulthood [1]. Also, as compared to general population, in which the incidence/probability of developing bipolar disorder in general population is 0.07, the probability of the same in patients with epilepsy is 1.69 cases per 1000 person-years, which is significantly high [2]. There have been reports of episodic attacks in chronic disorders: epilepsy and migraine. The diseases commonly occur together and share overlapping pathophysiological mechanisms and common clinical features. Recently identified common genetic markers and molecular substrates for epilepsy and migraine include mutations in genes like CACNA1A, ATP1A2, SLC1A3, and POLG. However, both conditions also have several distinct and important differences. Hence, the diagnosis and treatment of each of these diseases must take into consideration a potential presence of the other [3].

Keeping this in mind, we implement a strategic systems biology approach for structural and functional analysis of neurological protein interaction network. We aim to identify novel putative genetic markers through network analysis that could be the cause of comorbid conditions in neurological disorders. The approach followed for network analysis of neurological disorders in this study is unique and novel in several ways.

We have targeted the human neurological proteome for this study. Proteins function by interacting with one another and also with other molecules of the cell, like DNA and RNA, and mediate vital metabolic pathways, signalling cascades, cellular processes, and organismal systems. The unique function that each protein interaction confers to the system determines its affinity and specificity. Protein interactions therefore have a central role in the biological functioning of an organism and a perturbation of such interactions that might include gain of an inappropriate interaction or the loss of an important association controls the healthy and diseased state of an organism. Disease mutations affect the protein's binding interface causing biochemically dysfunctional allosteric changes in the protein's binding site. Studying protein interactions provides insights into the molecular basis of the disease, and this information can be used to devise better methods for prevention, diagnosis, and treatment of diseases [4].

The prioritization of novel candidates by machine learning in our study takes into consideration the structural descriptors of proteins in an interaction network. Machine learning techniques have been successfully used to find informative genes and mining critical information from raw data supplied to the machine. These prediction models have an increased interpretability and retain high accuracy to exploit the supplied data and figure out the required information effectively. Our work specifically deals with prioritizing novel gene products, that is, proteins that are previously not known to be associated with neurological disorders. This was done by identifying the characteristic protein network topological properties and 3-dimensional protein interface structural properties that are inherent in proteins that are known to be associated with neurological disorders. Protein interactions take place through protein interfaces. And differences in the structure of protein interface can lead to varied interactions. Hence, the structural properties of the protein interface play an important role in determining its interactions in a network. A similar study in which binding site structural characteristics were used to describe cancer associated human protein-protein interfaces in a cancer network shows that cancer protein interfaces have characteristic interface properties as compared with interfaces of normal proteins. Interface structural properties like accessible surface area (ASA), planarity, gap volume index, interface surface area, percent polar residues in the interface, percent nonpolar residues in the interface, and percent charged residues were used to describe cancer protein interfaces [5]. Our study used the same set of properties, calculated through the web tool 2P2I inspector (see Methodology), to characterize interfaces of proteins involved in neurological disease network. Two additional properties are used, namely, Gap Index and Interface Size, which are calculated from the above-mentioned seven properties.

To understand the global behaviour of proteins, the network graph representation with characteristic properties that define the participating proteins has been used previously. These are called network topological properties and include degree, betweenness, closeness, centrality, and shortest path length. The inclusion of protein interface structure and using network topological properties to describe their effect on the interacting proteins has been shown to identify essential hub proteins with better accuracy than before [6]. We have taken into consideration ten network topological properties that were calculated by the network analyser plugin of cytoscape (see Methodology).

Studies have shown that these protein descriptors (protein interface structural properties and network topological properties), individually, are optimal features of prioritizing novel candidates [7]. We have therefore considered both these structural descriptors together for the first time to describe and characterize known and novel potential neurological proteins under study. It was also observed that there was a decline in performance and accuracy of the classifiers at the cost of eliminating some of these features. And, therefore, all the 19 descriptors together were considered optimally fit to be included in the study.

Machine learning classifiers were trained on these parameters and subjected on known set of neurological gene products to identify their unique underlying patterns and relationship in a network and then use these to identify previously unknown markers of neurological disorders from a list of human proteins that are not known to be associated with such disorders. This structural level analysis provides important clues about the affinity and specificity of protein interactions, and hence only the proteins whose 3D structure is available were considered. Identified risk markers could be considered important to determine patient prognosis for neurological diseases. Identifying such markers by taking into consideration the properties of known genes and proteins involved in the disorders under study is known as gene prioritization [8].

Further, from the prioritized gene list, we identified proteins hubs that have the highest number of interacting partners in the network and can be thought to participate simultaneously in most neurological pathways.

## 2. Methodology

### 2.1. Screening of Genes under Study

Gene sets known to be associated with several neurological disorders, namely, epilepsy, Alzheimer's, Parkinson's, autism, schizophrenia, bipolar disorder, and migraine, were downloaded from Genotator (http://genotator.hms.harvard.edu/) [9], which is an online available real-time aggregation tool that has a multiquery engine. It automatically integrates data from 11 external clinical genetics resources to provide reliable ranking of genes in order of disease relevance and covers both historical genetics research and recent advancements and discoveries in disease genetics. Total number of unique genes associated with these disorders was 2,807. A list of 4,538 proteins corresponding to these genes was downloaded from Uniprot (http://www.uniprot.org/) [10]. For gene prioritization one of the parameters under consideration was interface structural properties of the interacting proteins in neurological disease network. To calculate the structural properties, the three-dimensional structures of all the proteins were downloaded from RCSB Protein Data Bank (PDB) (http://www.rcsb.org/) [11]. Therefore the PDB Id list for 4,538 proteins was extracted from a total number of 47,532 available human protein structures on PDB. 17,457 3D-protein structures corresponded to our list of 4538 proteins associated with the group of neurological diseases under study. This was considered as the known set of proteins (KSOPs). Remaining 30,075 protein structures from PDB were listed as the unknown set of proteins (USOPs), that is, the proteins not considered to be associated with the group of diseases under study. The list of 17,457 KSOPs included a number of structural variants associated with each protein. Hence this list was then manually sorted and only the protein structure with highest resolution was considered. Each protein chain structure was taken into account, as the protein structural interface that takes part in interacting with other proteins can be an assemblage of any combination of the available chains of a protein. Also, mutant and recombinant structures were avoided. If multiple structures were available for each chain, a single high resolution structure was considered specific to the protein chain. This is mainly because high resolution crystal structures of proteins enable understanding of their associated molecular mechanisms with high degree of precision and accuracy. Such accurate structures help in addressing biological questions of fundamental importance, as well as aiding in the study of drug designing and pharmacological research. Increase in the resolution of a structure increases the confidence about positions of atoms in the respective conformation. Accurate positions of atoms in a high resolution structure would help in specific and physiologically relevant interactions with other proteins in an interaction network. However, considering that high resolution has minimum effect on the errors arising from multiple conformations of the protein, therefore, taking the high resolution structure of all the conformations of each protein into consideration is important [12]. Excluding the independent chain structures would mean losing out information on any putative protein interface structure. This left us with a list of 2,487 KSOPs, each associated with its available chain structure. Similarly, 30,075 USOPs were sorted and screened for available high resolution chain structures of all proteins, and the final list reduced to 9,434 proteins. The unique set of proteins from 2,487 KSOPs and 9,434 USOPs was used for construction of a protein-protein interaction network that was further used to analyse network topological properties of these proteins. And, the entire set of 2,487 KSOPs and 9,434 USOPs was used to fetch the structural properties of each possible interface that could be formed by different combinations of protein chains for each protein.

### 2.2. Protein-Protein Interaction Network and Analysis of Protein Network Properties

881 and 4,073 unique set of total 2,487 KSOPs and 9,434 USOPs, respectively, were used as input for STRING (http://string-db.org/) to identify the potential protein-protein interactions that might have been predicted experimentally or computationally or published in literature [13]. A total of 683,159 interactions for all the proteins were extracted from STRING. The interactions were subsequently used as input to visualise and analyse a network using cytoscape (v 3.1.0) [14]. The final network had 4,954 nodes (corresponding to number of proteins in the network) and 683,159 edges (number of interactions between proteins). The network analyser, a well-known cytoscape plugin, was used to compute specific network topological properties of the protein-protein interaction (PPI) network [15]. The 10 network properties that were analysed for 4,954 proteins by this tool are listed in Table 1. The functional protein interactions extracted from STRING were also used as input for web based tool HUBBA (http://hub.iis.sinica.edu.tw/Hubba/index.php) that analyses potential hubs in the network [16]. Also, the set of 881 KSOPs was used as a separate input for STRING, and the resulting interactions were subject to HUBBA analysis, in order to identify known protein hubs, that is, the proteins that have the maximum number of interactions in the known neurological protein-protein interaction network.

### 2.3. Inclusion of Protein Interface Structural Properties

Protein-protein interactions take place through the binding site of the proteins contained in the protein interface. Multiple conformations of particularly the key residues in the binding sites make these interactions possible. However, in contrast to the numerous protein-protein interactions that are possible, there are only limited and specific binding site conformations that favour protein binding. Protein interface properties are therefore useful to describe the preference of protein interaction that could take place through that surface of the protein. A protein interface is formed from a couple of protein chains that provide the most favourable binding conformation to the protein. Only certain possible combinations of a couple of the number of protein chains associated with a single protein can form the protein interface. The list of favourable protein chain combinations that were involved in forming the interface of each protein was extracted from PIFACE (http://prism.ccbb.ku.edu.tr/piface/) [17]. The input for this included the PDB Id of the protein and any associated two protein chains at a time. PIFACE combinations were extracted for 2,487 PDB Ids corresponding to KSOPs and 9,434 PDB Ids corresponding to USOPs. The number of favourable protein chain combinations was 2,179 and 5,550 for KSOPs and USOPs, respectively. The favourable chain combinations were used for calculating interface structural properties, to be used as protein structural descriptors, with the help of an online available tool 2P2I inspector (http://2p2idb.cnrs-mrs.fr/2p2i_inspector.html) [18]. Input to this tool is a PDB Id and a combination of protein chains known to form a potential binding interface from PIFACE. The 9 protein structural descriptors that were assessed using 2P2I are listed in Table 1.

### 2.4. Gene Prioritization by Machine Learning

The network properties and interface properties for the KSOPs and USOPs were combined together to prepare files for machine learning using WEKA. Altogether there were 19 descriptors for all the proteins. WEKA (http://www.cs.waikato.ac.nz/ml/weka/) is a data mining software that includes a collection of machine learning algorithms [19]. For preparing the training file, the list of 2,179 KSOPs was divided into half after random shuffling. And the same number of protein entries was taken from the unknown set after randomly shuffling it. The resulting training file had 1,090 KSOPs and 1,090 USOPs. Various available algorithms from WEKA, like Bagging, Naïve Bayes, Random Forest, Rotation Forest, and K-star, were trained upon a specific set of features (network topological properties and interface descriptors) associated with the KSOPs using tenfold cross-validation. Building more than one model helps explain how different classifiers make varied predictions on the training set. The models predict the recall, ROC area, accuracy, precision, true positive rate and the false positive rate for the training set. The models were individually applied to the test file to identify proteins that had features similar to those of KSOPs. The test file included 1,089 remaining KSOPs and 4,460 USOPs. The model that made predictions on the test set with highest precision, recall, ROC area, and accuracy was considered as the best fit model. The predictions of this model were taken for further analysis.

The combined list of resulting putative neurological candidates from the test set and the list of KSOPs from the training and test sets were used as input for STRING to identify corresponding protein-protein interactions. These interactions were then used to identify hub proteins using HUBBA. This gave a list of important proteins with most interacting partners in the new network, which includes additional identified protein candidates.

The three identified hub protein lists are compared to understand the essentiality of common hubs in all the three protein networks, namely, human structural protein interaction network, neurologically associated structural protein interaction network, and the final network of known and putative neurologically associated proteins. DAVID (http://david.abcc.ncifcrf.gov/) analysis of hub protein list from the third network which includes neurologically associated and newly predicted protein candidates was performed, to identify common pathways between new candidates and existing neurological pathways [20, 21].

**Figure 1:**
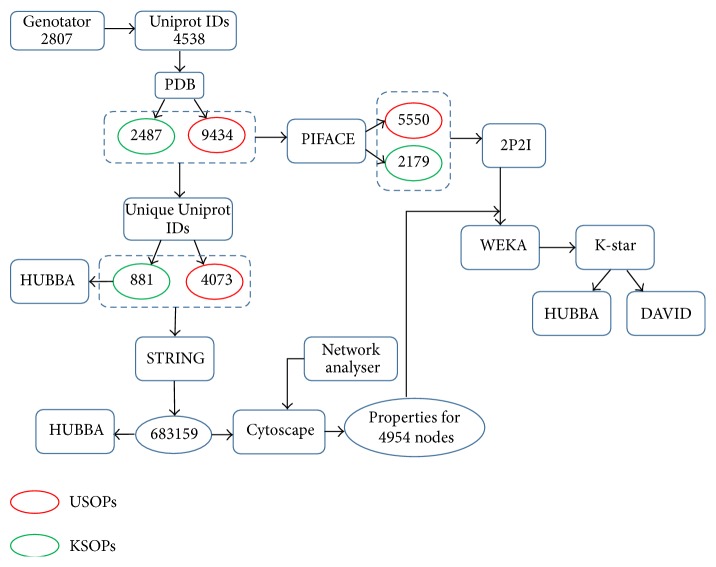
Flowchart of the methodology followed. KSOPs is the known set of proteins, and USOPs is the unknown set of proteins.

The strategic systems biology approach to identify novel neurological candidates followed by applying the above methodology also extends the machine learning predictions for analysing hubs protein candidates and has been illustrated in Figure 1.

## 3. Results and Conclusions

### 3.1. Results for Gene Prioritization by Machine Learning

**Figure 2:**
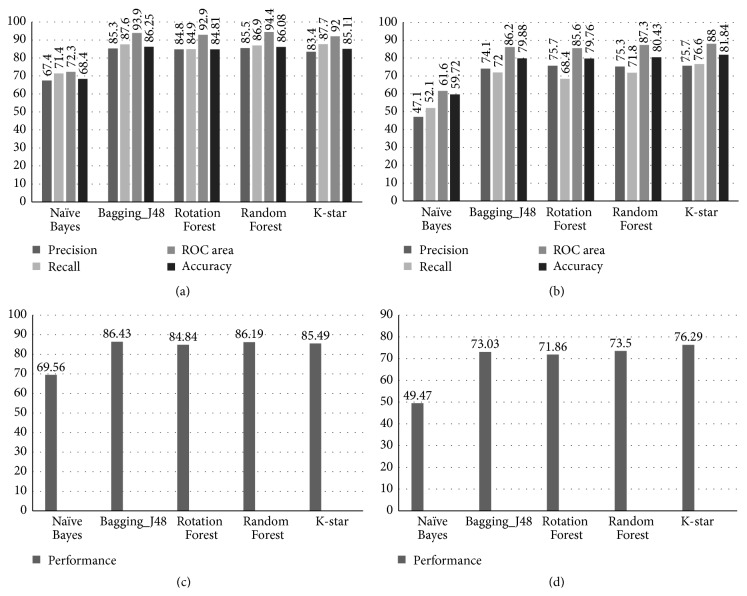
(a) Comparative representation of the performance metrics of five models for their predictions on the training dataset. (b) Comparative representation of the performance metrics of five models for their predictions on the test dataset. (c) Comparison of performance/*F*-score values for five models on training set. (d) Comparison of performance/*F*-score values for five models on test set.

WEKA classifiers were used to build models on the training set. The training set was built by random selection of 1,090 KSOPs and equal number of USOPs. Known proteins were the ones that are associated with neurological disorders. The unknown list consists of all other known human proteins whose 3-dimensional structures were available on the Protein Data Bank (PDB). There are 19 training features in both the training set and test set, for quantified description of the proteins. The features used for describing known proteins and evaluating novel candidates were an integration of protein network properties and interface structural properties. Five machine learning classifiers from WEKA were applied to the training set, to obtain five corresponding models. These include Naïve Bayes, Random Forest, Bagging with J48, Rotation Forest, and K-star. After 10-fold cross-validation, the predictions of the above classifiers on the training set are shown in Figure 2(a). Machine learning performance metrics show that the classifier Random Forest has the best predictions of precision, that is, the number of instances that have been predicted correctly as known proteins. Receiver operating characteristic (ROC) curve is a graph that illustrates the performance of a classifier as its discrimination threshold is varied; and Random Forest has the maximum area under ROC curve. Maximum recall and accuracy have been achieved by Bagging combined with J48. Sensitivity/recall is the number of known proteins that have been predicted correctly as being known. Accuracy is the proportion of true results, that is, the known proteins, and the unknown proteins classified as known. The performance of a single test/classifier prediction can be calculated using the precision and the recall. The *F*-score is a single measure of the performance of the prediction, where

(1) $$\begin{array}{c} F=2\left(\;\frac{PRECISION*RECALL}{PRECISION+RECALL}\;\right). \end{array}$$

The performance/*F*-score for all the classifiers on training sets were calculated by the above formula and are depicted in Figure 2(c) [22]. The graph shows that the classifier Bagging_J48 has the best performance.

The models that were built on training set after 10-fold cross-validation were applied on the test dataset individually to obtain the results. The following result predictors like precision, accuracy, recall, and area under ROC curve describe how successful the models have been to mine candidate proteins involved in neurological disorders, from a set of proteins that are previously not known to be associated with neurological disorders. The test file included 1,089 remaining KSOPs and 4,460 USOPs. Values of all the four performance metrics on the test set data for the 5 classifiers used are shown in Figure 2(b). Among the five models used for predictions on test set data, the K-star algorithm gives best result for precision, recall, and area under ROC curve as well as for the accuracy of predictions. Performance/*F*-score is calculated for all the models and is depicted in Figure 2(d). Different models might perform variedly depending upon the dataset and the information contained. For the present test set, the model that gave best results was K-star. However, it can be noted that Bagging_J48 has better overall performance on the training set, whereas K-star has the third best performance in the same dataset after Bagging_J48 and Random Forest. From these results it can be concluded that K-star algorithm makes better discretion of noise and signal, whereas other algorithms overfit in the present data lead to inclusion of random error/noise. K-star specifically learns the heterogeneity and relatedness/relationship of training set data more accurately than any other algorithm and gives the best performance in test set data by identifying maximum true positives.

The best predicted putative candidate proteins from the results of K-star algorithm are used for further analysis.  Table 2 shows the list of best 10 predictions of putative protein candidates from the unknown test set as obtained from the WEKA results of all five classifiers. Many putative candidate protein predictions were found to be common in all five model results. However, they have different prediction probabilities in all results. The fact that some proteins were commonly predicted by all the classifiers increases the probability of those proteins as potential candidates, as they got mined by all classifiers. Since, in certain classifier results, they might have lower prediction probabilities, they will not be taken as putative markers. Best prediction probabilities describe how accurately the previously not considered a neurological disorder candidate protein is predicted to be associated with neurological disorders.

### 3.2. Results from Hub Object Analyser

The Hub Object analyser (HUBBA) is a web based tool that finds hub proteins from the input protein interaction data. Hub proteins have characteristic greater number of interactions in a network than other non-hub proteins. In other words these proteins have more interaction partners, making them physiologically important for the individual. Hub proteins are essential elements and are indispensable for an individual's survival [23].

**Figure 3:**
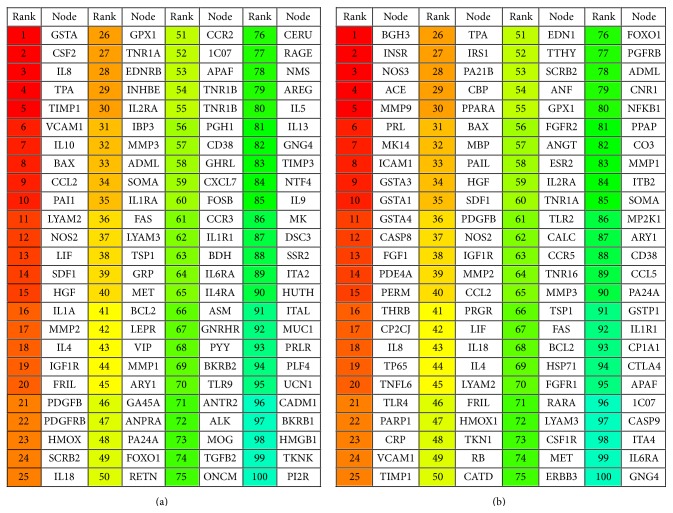
(a) Hub proteins identified in the human structural protein interaction network. (b) Hub proteins identified in the human neurological protein interaction network. The hubs are ranked in the decreasing order of priority.

The first set of analysed hub proteins is from the human protein interaction network. All the protein interactions that were used for hub identification include proteins partners that have an available 3D structure in PDB. The list of prioritised hubs for the same is shown in Figure 3(a).

**Figure 4:**
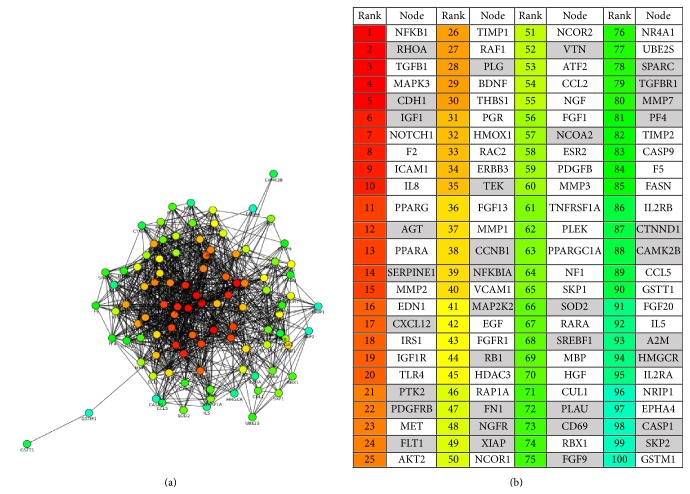
(a) Hub network of KSOPs and putative candidates. The proteins at the centre of the network are the ones that form most interactions with other proteins. (b) Hub proteins identified in the human neurological protein interaction network that includes newly identified gene products. The predicted gene candidates that are behaving as hubs have been highlighted.

The hubs are ranked on the basis of decreasing order of priority, marked by the color coding. We then find out how many of these hubs are common to hubs in the protein interaction network of neurological disorders, that is, the list of KSOPs involved in neurological disorders. The list of hub proteins from KSOPs is given in Figure 3(b). There are 21 neurologically important hub proteins that are present in the first 100 hub proteins' list of the human interaction network. The third hub proteins list is predicted from protein interaction data of known proteins and candidates prioritized by K-star algorithm of machine learning. K-star has been shown to predict novel neurological candidates with maximum accuracy; therefore the list of genes prioritized by this algorithm can be relied on with more confidence and has been used for prediction of hub proteins in the neurological network. Therefore, in addition to known neurological disease candidates, this list includes previously unknown putative neurological candidates. Their interaction data is extracted from STRING, and the same is used as input for HUBBA. This analysis informs if any of the previously unknown neurological candidates act as hub proteins in human neurological protein interaction network. The network and list of first 100 hub proteins from this analysis are shown in Figures 4(a) and 4(b), respectively. Out of these 100 protein hubs, 44 are the predicted putative neurological candidates by machine learning, which tend to play important role in neurological protein network and are highlighted in Figure 4(b).

**Figure 5:**
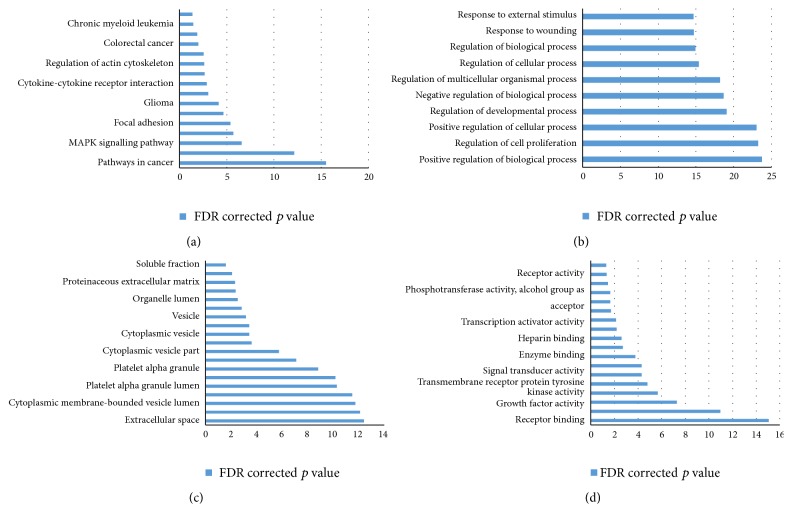
(a) Most enriched KEGG pathways from DAVID analysis. (b) Enriched biological processes. (c) Enriched cellular components. (d) Enriched molecular functions.

The identified 100 high ranking hubs from the third hub protein list were used as input for DAVID for pathway and gene ontology analysis. The most enriched pathways, biological processes, cellular components, and molecular functions are represented in Figure 5.

## 4. Discussion

The present study describes a structural systems biology approach for gene prioritization of novel protein candidates involved in neurological protein interaction networks. We have considered neurological disorders like epilepsy, Alzheimer's, Parkinson's, bipolar disorder, autism, migraine, and schizophrenia for building a neurological protein network. These disorders share symptoms like depression, cognitive impairment, and anxiety. Experimental studies have shown that aberrations in the same gene or genomic area could increase the risk of acquiring several complex neuropsychiatric disorders owing to the fact that there are a number of underlying mechanisms and complex pathways associated with multiple diseases [24]. The interplay of numerous specific and nonspecific risk factors that result in coexistence of psychiatric and medical conditions makes diagnosis and treatment difficult that often results in other abnormalities, as in case of schizophrenia [25].

Gene prioritization for neurological disorders is an important area of research. Several studies that identify putative candidates in disorders like autism and epileptic encephalopathy have been carried out [26, 27]. Scoring systems have been developed previously, using gene prioritization based on human population genetics data to identify common* de novo* functional variants across four neuropsychiatric disorders, namely, epileptic encephalopathies, severe intellectual disability, schizophrenia, and autism spectrum disorders. The authors in this paper have identified hot zone* de novo* mutations in these disorders that occur in the most intolerant genes [28].

In our study we use structural protein descriptors for prioritizing novel shared genetic markers in several neurological disorders owing to the specificity and characteristic structural properties of genes involved in similar phenotypic expression.

Exome sequencing studies on over thousand patient samples have identified the importance of* de novo* and gene-disruption events in neurological disorders. Deciding on potential candidates for study is dependent on many factors including recurrence of mutation and involvement of the gene in the disease protein network [29]. For example, recurrent microdeletion at the locus 1q21.1 has been associated with numerous phenotypic abnormalities including of the brain, the heart, and the eye. This emphasizes the relevance of a genotypic approach to clinically manage the treatment of different patients [30]. The method used for gene prioritization is machine learning, and the features that are used to train the machine are structural descriptors of proteins. Therefore, only the proteins whose 3D structure is available on the Protein Data Bank are used for the study. The structural descriptors include protein network properties and protein interface structural properties. Various studies have shown that the protein network topological properties and interface structural properties are essential features for protein prioritization [31]. This study for the first time integrates these both features for gene prioritization of novel neurological candidates. Five machine learning algorithms were used for protein prioritization by WEKA. These included Naïve Bayes, Rotation Forest, Random Forest, Bagging-j48, and K-star. Maximum accuracy of 81.84%, a precision of 88%, and recall of 76.6% were achieved by the K-star algorithm on the test set, making its predictions the most reliable to use for further study and analysis.

Previously also certain genetic markers and pathways have been identified that are shared in these disorders. However, a lot of scope remains. Our work identifies markers that are important in the network of these diseases. Also, previously unknown markers that might be involved in shared pathways of these neurological disorders have been identified using HUBBA. The hubs identified in the neurological disease network are the ones that have highest number of interactions in the network and therefore might be involved in multiple neurological pathways. DAVID analysis identifies the involvement of novel candidates in the existing neurological interaction network, wherein multiple signalling pathways like receptor binding, pathways in cancer, and cell proliferation, among others, get enriched. Previous studies also suggest that there are overlapping molecular pathways implicated in neurological disorders and cancers. Genes associated with these two groups of diseases are associated with kinase signalling, control of cell cycle, and cellular processes and DNA repair [32].

This comprehensive neurological disease protein network analysis has therefore identified significant candidates that could be responsible for existence of shared clinical manifestations between these diseases that makes diagnosis and treatment difficult and also leads to resistance to treatment in certain cases. We present a larger network view that takes care of multiple interactions and molecular pathways associated with such diseases of comorbid phenotype. Novel protein hubs that have been identified are important potential candidates for studying neurological disorders. The promising pattern of observations from this study and the procedure followed sets an example to conduct such more comprehensive analysis which can then be taken to next level of experimental validation.

## Figures and Tables

**Table 1:** Protein structural descriptors considered for machine learning.

Protein network properties	Protein interface structural properties
Average shortest path length	Total accessible surface area

Clustering coefficient	Gap volume

Closeness centrality	Average interface surface area

Eccentricity	Percent average neutral residues

Stress	Percent average polar residues

Degree	Percent average nonpolar residues

Betweenness	Percent average charged residues

Neighbourhood connectivity	Gap Index

Radiality	Interface Size

Topological coefficient	

**Table 2:** Ten best putative candidate predictions with a probability of 1, from all five machine learning algorithms used.

Naïve Bayes	Bagging-J48	Rotation Forest	Random Forest	K-star
1HRK	1S18	1ZT4	1CC0	1G82
1MR8	1J1J	1MR1	1CI4	2GJX
1WNT	1OPL	2K03	1CKS	1MR1
1Z6X	1WMH	1L9X	1CZZ	1NR4
2B2Y	2ARY	1I3O	1DZA	1ZSV
2J4E	1I3O	1X86	1GWQ	1S18
2NN6	2B5N	1XV9	1HLO	1WPQ
1H28	1H4O	1ZT4	1HYN	1CKS
1IYI	1YBO	2PO6	1IRJ	1NLW
1Z6U	2DSQ	2EWY	1KHU	1KN0

## References

[1] Stafstrom C. E., Hagerman P. J., Pessah I. N., Noebels J. L., Avoli M., Rogawski M. A. (2012). Pathophysiology of epilepsy in autism spectrum disorders. *Jasper's Basic Mechanisms of the Epilepsies*.

[2] Ettinger A. B., Reed M. L., Goldberg J. F., Hirschfeld R. M. A. (2005). Prevalence of bipolar symptoms in epilepsy vs other chronic health disorders. *Neurology*.

[3] Bianchin M. M., Londero R. G., Lima J. E., Bigal M. E. (2010). Migraine and epilepsy: a focus on overlapping clinical, pathophysiological, molecular, and therapeutic aspects. *Current Pain and Headache Reports*.

[4] Chen J., Sawyer N., Regan L. (2013). Protein-protein interactions: general trends in the relationship between binding affinity and interfacial buried surface area. *Protein Science*.

[5] Kar G., Gursoy A., Keskin O. (2009). Human cancer protein-protein interaction network: a structural perspective. *PLoS Computational Biology*.

[6] Gursoy A., Keskin O., Nussinov R. (2008). Topological properties of protein interaction networks from a structural perspective. *Biochemical Society Transactions*.

[7] Johnson M. E., Hummer G. (2013). Interface-resolved network of protein-protein interactions. *PLoS Computational Biology*.

[8] Zhang K. X., Ouellette B. F. F. (2011). CAERUS: predicting CAncER outcomes using relationship between protein structural information, protein networks, gene expression data, and mutation data. *PLoS Computational Biology*.

[9] Wall D. P., Pivovarov R., Tong M. (2010). Genotator: a disease-agnostic tool for genetic annotation of disease. *BMC Medical Genomics*.

[10] The UniProt Consortium (2015). UniProt: a hub for protein information. *Nucleic Acids Research*.

[11] Berman H. M., Westbrook J., Feng Z. (2000). The protein data bank. *Nucleic Acids Research*.

[12] Acharya K. R., Lloyd M. D. (2005). The advantages and limitations of protein crystal structures. *Trends in Pharmacological Sciences*.

[13] Szklarczyk D., Franceschini A., Wyder S. (2015). STRING v10: protein-protein interaction networks, integrated over the tree of life. *Nucleic Acids Research*.

[14] Shannon P., Markiel A., Ozier O. (2003). Cytoscape: a software environment for integrated models of biomolecular interaction networks. *Genome Research*.

[15] Assenov Y., Ramírez F., Schelhorn S.-E. S.-E., Lengauer T., Albrecht M. (2008). Computing topological parameters of biological networks. *Bioinformatics*.

[16] Lin C.-Y., Chin C.-H., Wu H.-H., Chen S.-H., Ho C.-W., Ko M.-T. (2008). Hubba: hub objects analyzer—a framework of interactome hubs identification for network biology. *Nucleic Acids Research*.

[17] Cukuroglu E., Gursoy A., Nussinov R., Keskin O. (2014). Non-redundant unique interface structures as templates for modeling protein interactions. *PLoS ONE*.

[18] Basse M. J., Betzi S., Bourgeas R. (2013). 2P2Idb: a structural database dedicated to orthosteric modulation of protein-protein interactions. *Nucleic Acids Research*.

[19] Hall M., Frank E., Holmes G., Pfahringer B., Reutemann P., Witten I. H. (2009). The WEKA data mining software: an update. *ACM SIGKDD Explorations Newsletter*.

[20] Huang D. W., Sherman B. T., Lempicki R. A. (2009). Systematic and integrative analysis of large gene lists using DAVID bioinformatics resources. *Nature Protocols*.

[21] Huang D. W., Sherman B. T., Lempicki R. A. (2009). Bioinformatics enrichment tools: paths toward the comprehensive functional analysis of large gene lists. *Nucleic Acids Research*.

[22] Sokolova M., Lapalme G. (2009). A systematic analysis of performance measures for classification tasks. *Information Processing & Management*.

[23] Batada N. N., Reguly T., Breitkreutz A. (2006). Stratus not altocumulus: a new view of the yeast protein interaction network. *PLoS Biology*.

[24] Zhu X., Need A. C., Petrovski S., Goldstein D. B. (2014). One gene, many neuropsychiatric disorders: lessons from Mendelian diseases. *Nature Neuroscience*.

[25] Cosoff S. J., Hafner R. J. (1998). The prevalence of comorbid anxiety in schizophrenia, schizoaffective disorder and bipolar disorder. *Australian and New Zealand Journal of Psychiatry*.

[26] Wall D. P., Esteban F. J., DeLuca T. F. (2009). Comparative analysis of neurological disorders focuses genome-wide search for autism genes. *Genomics*.

[27] Oliver K. L., Lukic V., Thorne N. P. (2014). Harnessing gene expression networks to prioritize candidate epileptic encephalopathy genes. *PLoS ONE*.

[28] Petrovski S., Wang Q., Heinzen E. L., Allen A. S., Goldstein D. B. (2013). Genic intolerance to functional variation and the interpretation of personal genomes. *PLoS Genetics*.

[29] Hoischen A., Krumm N., Eichler E. E. (2014). Prioritization of neurodevelopmental disease genes by discovery of new mutations. *Nature Neuroscience*.

[30] Mefford H. C., Sharp A. J., Baker C. (2008). Recurrent rearrangements of chromosome 1q21.1 and variable pediatric phenotypes. *The New England Journal of Medicine*.

[31] Linghu B., Snitkin E. S., Hu Z., Xia Y., Delisi C. (2009). Genome-wide prioritization of disease genes and identification of disease-disease associations from an integrated human functional linkage network. *Genome Biology*.

[32] Plun-Favreau H., Lewis P. A., Hardy J., Martins L. M., Wood N. W. (2010). Cancer and neurodegeneration: between the devil and the deep blue sea. *PLoS Genetics*.

